# A Personalized Mobile Cessation Intervention to Promote Smokers From the Preparation Stage to the Action Stage: Double-blind Randomized Controlled Trial

**DOI:** 10.2196/41911

**Published:** 2023-04-26

**Authors:** Haoxiang Lin, Ying Wang, Yanling Xing, Yinglian Han, Chengqian Zhang, Ting Luo, Chun Chang

**Affiliations:** 1 Institute for Global Health and Development Peking University Beijing China; 2 Linzi Disease Control and Prevention Center Zibo China; 3 School of Public Health Peking University Beijing China

**Keywords:** smoking cessation, mobile health, health education, smoking, behavior intervention, behavior change, support, text message, personalized, smoking abstinence, health behavior, health promotion

## Abstract

**Background:**

Most mobile cessation studies have found that such interventions have a higher quitting rate than interventions providing minimal smoking cessation support. However, why such interventions are effective has been almost unstudied by researchers.

**Objective:**

This paper describes the principles of the personalized mobile cessation intervention-based WeChat app and used generalized estimated equations to assess why a personalized mobile cessation intervention was more likely to promote smokers from the preparation stage to the action stage than a nonpersonalized intervention.

**Methods:**

This is a 2-arm, double-blind, randomized controlled trial in five cities in China. The intervention group received a personalized mobile cessation intervention. The control group received a nonpersonalized SMS text message smoking cessation intervention. All information was sent by the WeChat app. The outcomes were the change in protection motivation theory construct scores and the change in transtheoretical model stages.

**Results:**

A total of 722 participants were randomly assigned to the intervention or control group. Compared with those who received the nonpersonalized SMS text message intervention, smokers who received the personalized intervention presented lower intrinsic rewards, extrinsic rewards, and response costs. Intrinsic rewards were determinants of stage change, thus explaining why the intervention group was more likely to promote smokers from the preparation stage to the action stage (odds ratio 2.65, 95% CI 1.41-4.98).

**Conclusions:**

This study identified the psychological determinants at different stages to facilitate smokers moving forward to the next stage of quitting behavior and provides a framework to explore why a smoking cessation intervention is effective.

**Trial Registration:**

Chinese Clinical Trial Registry ChiCTR2100041942; https://tinyurl.com/2hhx4m7f

## Introduction

Smoking kills more than 1 million people in mainland China every year, and the number is predicted to grow to 2 million by 2030 and 3 million by 2050 [1]. The 2015 China Adult Tobacco Survey estimated that there were 316 million smokers (smoking prevalence for adults: 27.7%); although 18% were planning to quit smoking within 12 months [2], only a few could obtain effective smoking cessation services due to the imbalanced distribution of resources, limited availability of medications, and lack of specialist training for doctors [3]. Thus, additional efforts to identify universal, cost-effective, and personalized smoking cessation services are warranted in China and in most low-income countries worldwide.

A large number of studies have shown that mobile cessation interventions can improve the smoking cessation rate and have the potential to provide smoking cessation services broadly, but most previous studies have focused only on the quitting rate [4-6]. The other part, psychological change, has remained understudied. We recognize that the quitting rate is important, but given that many people fail in their attempts to quit smoking, measuring the quitting rate alone provides only a partial picture. We believe that it is important to measure psychological change and target behavior simultaneously.

Another dimension is the determinant of quitting behavior. Several recent studies have noted that some factors could be associated with short-term or long-term abstinence [7,8]. Few studies have treated smoking cessation as a movement process, and the modifiable psychological determinants at different stages to facilitate smoker movement from precontemplation to the action period have been almost entirely ignored by any previous researchers. As a result, once a mobile cessation intervention has been confirmed to be effective, researchers have no idea why such interventions are effective. This gap also leaves researchers unclear regarding how they can take advantage of previous experiences [9].

To help fill this evidence gap, we conducted a randomized controlled trial (RCT) to provide valuable evidence on these topics. We found that a personalized and behavior change theory–based SMS text message smoking cessation intervention was more effective than a nonpersonalized SMS text message intervention (6.9% vs 3.0%; adjusted odds ratio [OR] 2.66, 95% CI 1.21-5.83). The effectiveness of our intervention has been reported in *JAMA Network Open* [10].

The purpose of this study was to evaluate the changes in protection motivation theory (PMT) subconstructs and the transtheoretical model (TTM) stages of change to provide insight into why a personalized mobile cessation intervention was more likely to promote smokers from the preparation stage to the action stage. To our knowledge, this study is the first RCT using personalized SMS text messages for a mobile cessation intervention with a positive control group design in a country with a limited tobacco control policy.

The TTM has been widely used for health behavior change interventions. The theory identifies five stages of change: precontemplation, contemplation, preparation, action, and maintenance. It is an integrative behavior change model for intentional change focused on the decision-making of individuals. Given the systematic relationship between the stages and processes of change, several strategies have been used to strengthen behavior changes or to achieve the next stage [11,12]. The PMT is a famous theory of behavior change. The basic idea behind this theory is that prevention behavior is driven by an evaluation of the risk and coping response. It has seven elements: perceived severity, perceived susceptibility, intrinsic and extrinsic rewards, response efficacy, self-efficacy, and response costs [13,14]. Among the major theories currently used in the field of addiction, the PMT could be particularly well suited for understanding smoking behavior since it has a variable to explain the perceived positive effect of a negative behavior in its framework. A large number of studies have discussed the associations between PMT subconstructs and smoking behavior [15,16], but none of the studies used a prospective cohort study design to assess the relationships of PMT subconstructs with the stage of change for smokers in quitting smoking.

Many studies have found that personalized and behavior change theory–based interventions could be more effective than only providing broad, nonspecific advice [17,18] because self-relevant information can easily engage attention, increase information processing motivation, and cause more behavioral changes [18]. Most personalized communications are achieved through ongoing evaluation; if smokers join the information generation process, it can also increase compliance motivation. Therefore, we posit our first hypothesis: a personalized and behavior change theory–based SMS text message smoking cessation intervention can provide more positive psychological change (PMT subconstructs and TTM stage of change) than a nonpersonalized SMS text message intervention.

Our previous research used cross-sectional data and found that smokers have different psychological factors that influence their quitting intention or behavior [15]. For example, perceived vulnerability, self-efficacy, response efficacy, and intrinsic rewards were associated with motivating smokers’ quitting intentions, and intervention activities focusing on those points were appropriate for smokers in the precontemplation and contemplation stages. However, only self-efficacy and response costs were associated with promoting smokers to have actual quitting behaviors, and intervention activities focusing on these points are appropriate for smokers who are in the preparation stage. Therefore, we posit our second hypothesis: the psychological determinants at different stages to facilitate smoker movement from precontemplation to the action period vary.

## Methods

### Design and Inclusion

We conducted a 2-arm, double-blind, RCT in five cities in China (Beijing, Dezhou, Baotou, Yakeshi, and Linzi). Participants were randomized to an intervention group or a control group between April 2021 and July 2021. Daily or weekly smokers 18 years or older who smoked more than 5 cigarettes per week were eligible for inclusion if they owned a mobile phone and used WeChat. We advertised the trial to smokers through paper advertisements (leaflets), digital advertisements (WeChat), and staff (teachers and leaders). Potential participants contacted the local Center for Disease Control and Prevention to register. All eligible smokers were told they needed to come to a specific place on a fixed date to finalize the recruitment process. Participants’ eligibility was double-checked, and they signed an informed consent form during the first face-to-face contact.

### Ethics Approval

The trial was approved by the Ethics Committee of Peking University Health Science Center (IRB00001052-30063). All the participants signed informed consent forms before randomization and knew that they could withdraw from the study at any time. All patients’ information was accessible only to the personnel participating in the study. We did not provide money to the participants, but we provided gifts (a towel, an umbrella, or a cup) if the participants completed one follow-up visit. The clinical trial registration number is ChiCTR2100041942.

### Development of the Text Bank

The first stage was to develop the intervention message bank. Messages were developed by Peking University, School of Public Health, with the input of smokers and smoking cessation professionals. The messages had a three-layer framework. The first layer was divided based on time and consisted of the prequit message (1-7 days), quit day message (8 days), withdrawal symptom management message (9-18 days), early quit message (19-36 days), and late period message (37-90 days). The second layer was divided based on the TTM. Before the quit day, messages were classified as strong quitting intention or weak quitting intention. After the quit day, messages were classified as maintained abstinence or relapsed. The third layer was divided based on the PMT. Messages were classified as increased severity and susceptibility, decreased response cost and intrinsic and extrinsic rewards, and increased self-efficacy and response efficacy. The core motivational messages consisted of 14 subgroups with a total of 200 SMS text messages. There were also approximately 200 contact messages. The participants received partial information, and the information they received was different depending on their status. The WeChat app evaluated smokers’ status on day 0, day 19, day 36, day 45, day 60, and day 75 to decide what messages to send them.

### Study Instruments

All messages were sent through the WeChat app (the most popular Chinese mobile chatting app) [4]. The WeChat app evaluated the PMT construct score by asking questions and recording information; then, it automatically calculated the lower score of the subcontent that needed to be strengthened. Specifically, the scale comprised 21 items using a 7-point Likert-type scale with responses ranging from 1 (definitely disagree) to 7 (definitely agree). Each construct subscale included three items, and we computed the mean as the subscale score. We have published the details of this scale and evaluation process elsewhere [15-18]. To evaluate smokers’ quitting intention, we used a 5-point scale ranging from not at all likely (1) to very likely (5) regarding the likelihood that they would try to quit in the next 6 months (1-3: weak intention; 4-5: strong intention) [19]. A more detailed framework of the text and intervention based on the TTM and PMT is reported separately [5,10].

The last stage was to deploy the message library on the WeChat platform by using IT. Our IT team completed the development process with several important considerations. First, the app needed to ensure the confidentiality of the data. Second, the system needed to be user-friendly with ease for quick data entry. Third, there needed to be a back-end server to store the data. Fourth, the information needed to be presented in a way that could be easily read and interpreted.

### Randomization and Blinding

After recruitment, the participants were required to complete the baseline questionnaire and register through WeChat. A randomized block design was used, and the score of the Fagerström Test for Nicotine Dependence was treated as a stratified factor. The WeChat system automatically generated two blocks by the score of the test: (1) low or moderate nicotine dependence group (0-6 points) and (2) high dependence group (≥7 points) [20]. Eligible participants were assigned to the intervention group or the control group within each block by simple randomization. Independent IT personnel created a randomization sequence but were not involved in the research. The WeChat system was also used to balance demographic characteristics. Randomization was fully computerized and automated with equal allocation. The researchers and participants were all blinded.

### Intervention and Control Group

All participants were informed that the eighth day after randomization would be their quit day. Participants who were allocated to the intervention group received the program of interventions. As described above, this consisted of 1-2 personalized SMS text messages per day for 3 months.

Control group participants received a nonpersonalized SMS text message smoking cessation intervention developed by the National Cancer Institute. It was based on well-established cognitive behavioral cessation approaches and contained 91 messages. Participants received approximately 1 message per day for 3 months. The SMS text messages provided encouragement, practical advice to help maintain cessation, and information on the health effects of smoking. The details of the control group can be found elsewhere [7].

### Measures

All participants in the two groups were instructed to attend face-to-face follow-ups with research staff 1 month, 3 months, and 6 months after randomization. The outcomes were the change in PMT construct scores and the change in TTM stages. TTM stages of change were measured by smoking status using the question “Are you going to quit smoking?”: “No, not thinking of quitting” (precontemplation; individual is not ready or thinking about making a change); “Yes, within the next 6 months” (contemplation; individual thinking about making a change but not in the immediate future); “Yes, within the next 30 days” (preparation; individual ready to change, intends to try to change in the immediate future, and might be making small preparatory changes); and action, the biochemically verified patient has not smoked since the last follow-up [21,22].

### Statistical Analysis

#### Sample Size Calculation

The sample size calculation was based on the formula for a 2-arm RCT. Based on earlier research, we estimated that biochemically verified continuous smoking abstinence at 6 months would be approximately 4% in the control group and 10% in the intervention group [1,4,7]. To achieve 80% power with a significance level of .05 (2-sided), a sample size of 280 individuals was needed in each group. Assuming 20% attrition in the follow-up measurements, the total required sample size was 672.




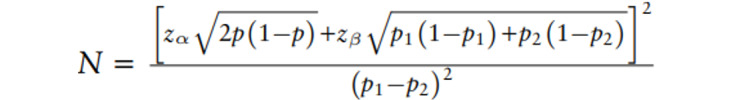




#### Data Analysis

Our data analysis was conducted in three steps. First, we used generalized estimating equations (GEEs) to analyze the changes in TTM stages by group. Second, we analyzed the associations between PMT construct scores and the change in TTM stages. Third, we assessed PMT construct score changes in the two groups to explore why a personalized mobile cessation intervention was more likely to promote smokers from the preparation stage to the action stage. A *P* value <.05 was considered significant. All statistical analyses were conducted using SPSS software, version 19.0 (IBM Corp). We performed an intention-to-treat analysis for each group.

#### Step 1

A GEE was used to address the correlated nature of the repeated measures in the data [20]. We estimated the impact of the intervention on the TTM stage of change in the first set of models. The dependent variables were TTM stages (if moving forward to the next stage=1; otherwise=0). The explanatory variable was the group (intervention group=1; control group=0). We added an interaction term between time and group to show how the intervention effect changed with time. We controlled for education, alcohol consumption, age, chronic disease, and nicotine dependence in the analyses. We chose an exchangeable correlation structure for the matrix structure and binary logistic regression for the model setting. The results are presented as ORs and 95% CIs.

#### Step 2

To examine the psychological determinants of movement from the precontemplation to the action period, we changed the explanatory variables to PMT construct scores. We used the second GEE to assess which PMT constructs were associated with the TTM stage of change. We chose an exchangeable correlation structure for the matrix structure and binary logistic regression for the model setting.

#### Step 3

We estimated the impact of the intervention on PMT construct scores in the last set of models to explore the reasons why the intervention group was more likely to promote smokers from the preparation stage to the action stage. The dependent variables included seven PMT constructs (severity, vulnerability, intrinsic rewards, extrinsic rewards, self-efficacy, response efficacy, and response cost). The explanatory variable was the group (intervention group=1; control group=0). We chose an exchangeable correlation structure for the matrix structure and linear regression for the model setting.

## Results

**Figure 1:**
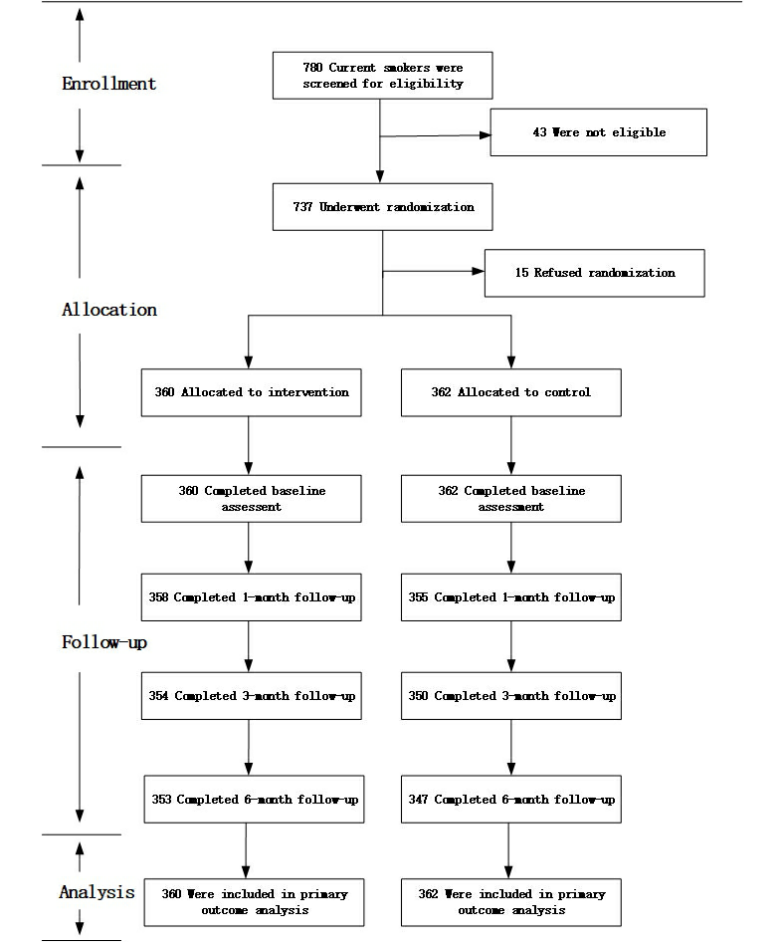
Trial profile. Note: According to the Russell Standard [21], those who declined to be involved in subsequent data collection were counted as smokers; therefore, cessation results were available for all 722 participants.

A total of 780 smokers were screened, and 722 (92.6%) participants were randomly assigned to the intervention or control group (Figure 1). We excluded 43 potential participants who were not eligible. All the participants were Chinese (Beijing: n=248, 34.3%; Dezhou: n=271, 37.5%; Baotou: n=99, 13.7%; Linzi: n=64 8.9%; and Yakeshi: n=40, 5.5%). All the treatment groups were well balanced with respect to baseline characteristics (Multimedia Appendix 1).

Table 1 shows the results of GEE estimation for the changes in the TTM stages. As shown, we found significant interactions between time and group for the preparation to action stages. This outcome suggests that the personalized intervention was more likely to promote smokers from the preparation stage to the action stage (OR 2.65, 95% CI 1.41-4.98). However, we did not find smokers in the intervention group more likely to move from the precontemplation to the contemplation stage or from the contemplation to the preparation stage.

**Table 1:** Generalized estimating equations estimation for change of transtheoretical model stages.

Variables^a^		Precontemplation to contemplation, OR^b^ (95% CI)	Contemplation to preparation, OR (95% CI)	Preparation to action, OR (95% CI)
**Group**				
	Control	Reference	Reference	Reference
	Intervention	1.34 (0.71-2.52)	0.85 (0.37-1.96)	0.83 (0.42-1.66)
**Time**				
	Month 1	Reference	Reference	Reference
	Month 3	0.74 (0.39-1.38)	0.71 (0.34-1.49)	0.98 (0.96-1.01)
	Month 6	0.75 (0.38-1.49)	0.40 (0.18-0.91)*	0.96 (0.64-1.46)
**Group × time**				
	Control	Reference	Reference	Reference
	Intervention × month 3	1.35 (0.54-3.36)	1.02 (0.34-3.05)	0.98 (0.94-1.02)
	Intervention × month 6	0.59 (0.22-1.57)	1.85 (0.59-5.82)	2.65 (1.41-4.98)*

^a^Control variables: education, alcohol drinking, age, chronic disease, and nicotine dependence.

^b^OR: odds ratio.

*P<.05

Table 2 shows the psychological determinants of movement from precontemplation to the action period. Self-efficacy (OR 1.20, 95% CI 1.03-1.39) and response efficacy (OR 1.30, 95% CI 1.04-1.64) were positively associated with smokers’ movement from the precontemplation stage to the contemplation stage. Only self-efficacy (OR 1.60, 95% CI 1.30-1.97) was positively associated with smokers’ movement from the contemplation to preparation stage. For the preparation to action stages, intrinsic rewards of smoking (OR 0.87, 95% CI 0.80-0.94) negatively influenced stage movement, and self-efficacy (OR 1.16, 95% CI 1.03-1.30) still had a positive influence.

Table 3 shows the results of GEE estimation for the PMT subconstructs. As shown, we found significant interactions between time and group for some subconstructs, suggesting that the intervention group showed significantly lower scores for the subconstructs of intrinsic rewards (OR 0.73, 95% CI 0.56-0.96) and extrinsic rewards (OR 0.71, 95% CI 0.55-0.90) of smoking and had a lower response cost of quitting (OR 0.75, 95% CI 0.57-0.99). These positive effects were stronger and more significant 3 months after the intervention.

Our analysis suggests that, compared with those who received the nonpersonalized SMS text message intervention, smokers who received the personalized intervention presented lower intrinsic rewards, extrinsic rewards, and response costs. Among these outcomes, intrinsic rewards were determinants of stage change (from the preparation stage to the action stage), thus explaining why the intervention group was more likely to promote smokers from the preparation stage to the action stage.

**Table 2:** Generalized estimating equations assessing the association of PMT subconstructs with moving forward in the stage of change.

Variables			Precontemplation to contemplation, OR^a^ (95% CI)		Contemplation to preparation, OR (95% CI)		Preparation to action, OR (95% CI)
**PMT^b^ subconstructs**							
	Perceived severity	1.29 (0.99-1.69)		0.89 (0.67-1.17)		0.92 (0.78-1.10)	
	Perceived vulnerability	0.95 (0.77-1.18)		1.14 (0.90-1.43)		1.03 (0.93-1.14)	
	Extrinsic rewards	0.94 (0.77-1.16)		0.98 (0.75-1.28)		1.03 (0.94-1.13)	
	Intrinsic rewards	0.89 (0.74-1.07)		0.83 (0.67-1.02)		0.87 (0.80-0.94)*	
	Self-efficacy	1.20 (1.03-1.39)*		1.60 (1.30-1.97)*		1.16 (1.03-1.30)*	
	Response efficacy	1.30 (1.04-1.64)*		1.09 (0.84-1.41)		1.11 (0.97-1.27)	
	Response cost	1.05 (0.88-1.24)		1.10 (0.88-1.37)		1.02 (0.92-1.14)	
**Age (years)**							
	18-44	Reference		Reference		Reference	
	45-64	0.94 (0.53-1.69)		1.32 (0.74-2.33)		0.55 (0.30-1.01)	
	>64	1.15 (0.23-5.66)		1.32 (0.27-6.60)		0.86 (0.13-5.81)	
**Nicotine dependence**							
	Low	Reference		Reference		Reference	
	Moderate	1.13 (0.63-2.00)		1.37 (0.78-2.39)		0.47 (0.22-1.02)	
	High	1.58 (0.64-3.90)		1.20 (0.44-3.27)		0.34 (0.07-1.66)	
**Education**							
	Middle school/lower	Reference		Reference		Reference	
	High school	0.91 (0.40-2.09)		0.65 (0.32-1.32)		1.31 (0.48-3.54)	
	College/above	0.70 (0.31-1.56)		0.45 (0.23-0.87)*		1.46 (0.58-3.65)	
**Chronic disease**							
	Have not	Reference		Reference		Reference	
	Have	0.62 (0.33-1.17)		1.19 (0.66-2.16)		1.05 (0.54-2.07)	
**Alcohol drinking**							
	Do not drink	Reference		Reference		Reference	
	Every month	1.43 (0.72-2.82)		1.02 (0.53-2.00)		0.78 (0.38-1.62)	
	Every week	1.68 (0.93-3.01)		0.81 (0.43-1.52)		0.71 (0.36-1.39)	
	Every day	5.79 (1.32-25.42)*		0.84 (0.34-2.07)		2.46 (0.67-9.00)	

^a^OR: odds ratio.

^b^PMT: protection motivation theory.

*P<.05

**Table 3:** Generalized estimating equations estimation for protection motivation theory subconstructs.

Variables^a^			Perceived severity, OR^b^ (95% CI)		Perceived vulnerability, OR (95% CI)		Intrinsic rewards, OR (95% CI)		Extrinsic rewards, OR (95% CI)		Self-efficacy, OR (95% CI)		Response efficacy, OR (95% CI)		Response cost, OR (95% CI)
**Group**															
	Control	Reference		Reference		Reference		Reference		Reference		Reference		Reference	
	Intervention	1.07 (0.90-1.28)		1.01 (0.81-1.24)		1.04 (0.81-1.34)		1.06 (0.85-1.31)		1.00 (0.79-1.27)		1.12 (0.91-1.38)		0.93 (0.73-1.18)	
**Time**															
	Month 0	Reference		Reference		Reference		Reference		Reference		Reference		Reference	
	Month 1	0.92 (0.79-1.07)		0.92 (0.78-1.07)		0.66 (0.55-0.80)*		0.78 (0.66-0.92)*		0.94 (0.77-1.15)		1.00 (0.84-1.20)		0.85 (0.70-1.02)	
	Month 3	0.92 (0.79-1.08)		0.83 (0.71-0.98)*		0.61 (0.50-0.74)*		0.81 (0.68-0.96)*		0.96 (0.79-1.18)		1.07 (0.89-1.28)		0.83 (0.67-1.02)	
	Month 6	0.96 (0.82-1.12)		0.89 (0.75-1.05)		0.56 (0.46-0.69)*		0.79 (0.66-0.95)*		1.07 (0.87-1.31)		1.19 (1.00-1.41)		0.71 (0.58-0.86)*	
**Group × time**															
	Control	Reference		Reference		Reference		Reference		Reference		Reference		Reference	
	Intervention × month 1	1.13 (0.91-1.39)		1.05 (0.83-1.33)		0.80 (0.61-1.05)		0.91 (0.72-1.15)		1.18 (0.91-1.54)		1.18 (0.93-1.49)		1.02 (0.78-1.32)	
	Intervention × month 3	1.21 (0.98-1.50)		1.14 (0.91-1.43)		0.73 (0.56-0.96)*		0.71 (0.55-0.90)*		1.26 (0.95-1.65)		1.25 (0.97-1.61)		0.75 (0.57-0.99)*	
	Intervention × month 6	1.21 (0.98-1.49)		1.09 (0.86-1.39)		0.80 (0.61-1.06)		0.68 (0.53-0.87)*		1.15 (0.87-1.53)		1.09 (0.86-1.40)		0.99 (0.75-1.30)	

^a^Control variables included age, education, living area, nicotine dependence, and alcohol drinking.

^b^OR: odds ratio.

*P<.05

## Discussion

### Principal Findings

This study engages in the debate in recent years over the effects of mobile cessation interventions. It provides a more comprehensive picture of the impact of personalized mobile cessation interventions from psychological change perspectives using Chinese data. It successfully shows that the intervention group had lower scores for the subconstructs of intrinsic rewards of smoking, extrinsic rewards of smoking, and response cost of quitting, and was more likely to promote smokers’ movement from the preparation stage to the action stage, confirming that personalized interventions can provide more positive psychological changes than a nonpersonalized SMS text message intervention. We believe that this finding is worth noting, as we extend the findings from target behavior to psychological change (first hypothesis).

Regarding the second hypothesis, although we found that the psychological determinants varied at different stages to facilitate smokers’ movement from precontemplation to the action period, self-efficacy was the most significant variable associated with forward movement in each stage of change. Self-efficacy is the belief in one’s competence to manage adversity in specific demanding situations. A branch of the literature has suggested that self-efficacy plays an important role in determining health behavior; for example, a German study found that self-efficacy was the strongest factor that signiﬁcantly predicted subsequent smoking-related behavioral intention [23]. This finding was also confirmed by other health behavioral studies. A meta-analysis reviewed 65 studies and suggested that coping appraisal variables and especially self-efficacy were the strongest predictors of protection motivation and behavior [24].

Another finding of our study is the identification of the psychological determinants at different stages to facilitate smokers moving forward to the next stage. The comparison between groups showed that the personalized intervention decreased the intrinsic rewards of smoking; this outcome is worth noting since it is a determinant of the movement from the preparation stage to the action stage. It also provides some indications of why a personalized intervention is more likely to promote smokers from the preparation stage to the action stage than a nonpersonalized intervention. Conversely, we found that our intervention did not lead to a significant change among smokers in the determinants that promoted smokers in moving forward from the precontemplation stage (self-efficacy and response efficacy) or contemplation stage (self-efficacy) to the next stage compared with the control group. This finding explains why our intervention had no better effect on promoting smokers to move forward from the precontemplation or contemplation stage, providing further direction for scaling up our intervention.

As stated, this RCT is the first to use personalized SMS text messages for a mobile cessation intervention with a positive control group design in China. We believe that our study has policy and theoretical implications. First, we identified psychological determinants of forward movement in the stage of change, which could be an important reference for further intervention. Second, this study demonstrated a clear framework for the intervention through the systematic and transparent application of the PMT and the TTM. It not only allows other researchers to take advantage of our experience when designing mobile interventions but also provides a framework for exploring why such an intervention is effective.

This study still has some limitations that could be addressed by further studies. First, although efforts were made to ensure that both the researchers and participants remained masked to the allocation, a risk of breaking the blinding was present. Second, some researchers believe that the deﬁnitions used for the TTM stages are arbitrary and that the categories are not qualitatively distinct [25,26]. Third, the scale that we used to evaluate PMT subconstructs was the same at each follow-up time; although there were intervals of a few months, some participants might have remembered some of the questions, providing inaccurate answers.

### Conclusion

This study reported clearly on the development of a mobile cessation intervention. GEE analysis identified the psychological determinants of forward movement in the stage of change and confirmed that a personalized mobile cessation intervention was more likely to promote smokers from the preparation stage to the action stage. The results of this empirical analysis could not only be equally applicable to the development of interventions targeting other health behaviors but also could provide a framework to explore why such an intervention is effective, thereby adding to earlier research on this topic.

## Appendix

Multimedia Appendix 1 Supplementary Table 1.

Multimedia Appendix 2 CONSORT checklist.

## Abbreviations

GEE: generalized estimating equation
OR: odds ratio
PMT: protection motivation theory
RCT: randomized controlled trial
TTM: transtheoretical model

## Data Availability

The data of the studies is accessible through the Peking University School of Public Health.
