# Supplementary material for: A Personalized Mobile Cessation Intervention to Promote Smokers From the Preparation Stage to the Action Stage: Double-blind Randomized Controlled Trial
Source: J Med Internet Res. 2023 Apr 26;25:e41911. doi: 10.2196/41911 (PMC10173036; doi:10.2196/41911)
Supplement: Multimedia Appendix 1 [file jmir_v25i1e41911_app1.docx]

**Supplemental Table 1. Characteristics of the Participants at Baseline**

|  |  | Control group  (n=362) [n(%)] | Intervention group（n=360）[n(%)] | | Total |
| --- | --- | --- | --- | --- | --- |
| Sex | Male | 358 (98.9) | | 358 (99.4) | 716(99.2) |
|  | Female | 4 (1.1) | | 2 (0.6) | 6 (0.8) |
| Age | 18-44 | 212 (58.6) | | 184 (51.1) | 396 (54.8) |
|  | 45-64 | 143 (39.5) | | 168 (46.7) | 311 (43.1) |
|  | ＞64 | 7 (1.9) | | 8 (2.2) | 15 (2.1) |
| Education | Middle school/less | 54 (14.9) | | 58 (16.2) | 112 (15.5) |
|  | High school | 73 (20.2) | | 96 (26.7) | 169 (23.4) |
|  | College/more | 235 (64.9) | | 205 (57.1) | 440 (61.0) |
| Ethnicity | Han | 341 (94.2) | | 336 (93.3) | 677 (93.8) |
|  | Other | 21 (5.8) | | 24 (6.7) | 45 (6.2) |
| Living area | Urban | 230 (65.9) | | 223 (62.8) | 453 (64.3) |
|  | Rural | 119 (34.1) | | 132 (37.2) | 251 (35.7) |
| Smoking frequency | Daily smoker | 293 (81.4) | | 299 (83.1) | 592 (82.2) |
|  | Weekly smoker | 67 (18.6) | | 61(16.9) | 128 (17.8) |
| Monthly income | ＜4000 Yuan | 177 (48.9) | | 169 (46.9) | 346(47.9) |
|  | 4000-5999 Yuan | 115 (31.8) | | 116 (32.2) | 231 (32.0) |
|  | ≥6000 Yuan | 70 (19.3) | | 75 (20.8) | 145 (20.1) |
| ND | Low | 228 (63.3) | | 228 (63.9) | 456 (63.6) |
|  | Moderate | 101 (28.1) | | 101(28.3) | 202 (28.2) |
|  | High | 31 (8.6) | | 28 (7.8) | 59 (8.2) |
| Have chronic disease | Yes | 92 (25.4) | | 98 (27.2) | 190 (26.3) |
|  | No | 270 (74.6) | | 262 (72.8) | 532 (73.7) |

ND: Nicotine dependence
